# Supplementary material for: Critical Parameters in an Enzymatic Way to Obtain the Unsweet Lactose-Free Milk Using Catalase and Glucose Oxidase Co-Encapsulated into Hydrogel with Chemical Cross-Linking
Source: Foods. 2022 Dec 26;12(1):113. doi: 10.3390/foods12010113 (PMC9818303; doi:10.3390/foods12010113)
Supplement: Supplementary file 1 [file foods-12-00113-s001.zip › foods-2042346-supplementary.pdf]

Supplementary Materials

Table S1. Glucose conversion yield [%] during one-pot biocatalysis (GOX- glucose oxidase from *Aspergillus niger*, CAT - the recombinant catalase preparation isolated from psychrotolerant bacteria *Serratia* sp.. glucose 27.5 g/L, 0.1M HEPES buffer pH 6.6, 12°C, $CaCO_3$ 4 g/L, without aeration).

GOX [g/L]	CAT [g/L]	Glucose conversion [%]		
		Time [hours]		
		**1.33**	**4**	**7.9**
0.6	0.2	18.51	25.01	38.88
1.2	0.2	25.06	29.14	44.64
2	0.2	29.77	33.52	46.73
3	0.2	35.92	38.12	52.09
4	0.2	38.81	40.97	55.6
0.6	0.6	21.47	42.06	55.66
1.2	0.6	26.37	43.9	57.49
2	0.6	35.97	47.85	62.58
3	0.6	37.41	49.12	64.37
4	0.6	40.9	52.14	68.11
0.6	1	24.24	45.23	59.19
1.2	1	29.9	48.67	62.27
2	1	34.09	51.94	68.05
3	1	39.68	56.32	71.32
4	1	39.85	67.91	86.21
6	1	44.68	72.56	89.93
8	1	48.81	75.05	90.68
6	1.2	42.27	72.21	84.94
8	1.2	50.96	79.09	90.98
